# Antioxidants of Natural Plant Origins: From Sources to Food Industry Applications

**DOI:** 10.3390/molecules24224132

**Published:** 2019-11-15

**Authors:** Sofia C. Lourenço, Margarida Moldão-Martins, Vítor D. Alves

**Affiliations:** LEAF, Linking, Landscape, Environment, Agriculture and Food, Instituto Superior de Agronomia, Universidade de Lisboa, Tapada da Ajuda, 1349-017 Lisboa, Portugal; sofiaclourenco@isa.ulisboa.pt (S.C.L.); mmoldao@isa.ulisboa.pt (M.M.-M.)

**Keywords:** natural antioxidants, extraction methods, stabilization techniques, food products

## Abstract

In recent years, great interest has been focused on using natural antioxidants in food products, due to studies indicating possible adverse effects that may be related to the consumption of synthetic antioxidants. A variety of plant materials are known to be natural sources of antioxidants, such as herbs, spices, seeds, fruits and vegetables. The interest in these natural components is not only due to their biological value, but also to their economic impact, as most of them may be extracted from food by-products and under-exploited plant species. This article provides an overview of current knowledge on natural antioxidants: their sources, extraction methods and stabilization processes. In addition, recent studies on their applications in the food industry are also addressed; namely, as preservatives in different food products and in active films for packaging purposes and edible coatings.

## 1. Introduction

Oxygen is an essential chemical element in the metabolism of aerobic organisms. However, it may trigger unfavorable reactions, and there has been a growing interest in studying the role of its reactive species. Reactive oxygen species (ROS) include free radicals like the superoxide anion, singlet oxygen, lipid peroxides and the hydroxyl radical. These reactive species are by-products of the normal cellular energy production and functional activities, presenting an important role in cell signaling, apoptosis, gene expression and ion transportation. Nevertheless, if ROS levels increase intensely, it can result in damage of many molecules, including proteins, lipids, RNA and DNA, since they are highly reactive. Furthermore, the production of free radicals is not only associated with the normal metabolic processes in the human body (endogenous sources), but can also be due to environmental factors (exogenous sources) such as stress, ozone radiation, pollution, pesticides and industrial chemicals [1,2,3,4,5,6]. When higher production of ROS in relation to their removal by biological systems (antioxidant defenses) occurs, it is called oxidative stress [7]. That has long been associated with increased risk for several diseases, such as cancer [6,8], diabetes, arthrosclerosis [7], arthritis [9], neurodegenerative diseases [10] and premature aging [11].

Antioxidants may protect cells by a variety of mechanisms, including the conversion of ROS to non-radical species (which are dependent on the antioxidant involved), breaking the auto-oxidative chain reaction initiated by ROS and decreasing localized oxygen concentrations [12,13]. The intake of exogenous antioxidants, such as ascorbic acid (Vitamin C), α-tocopherol (Vitamin E), carotenoids and polyphenols, that can be found in commonly consumed fruits, vegetables, beverages, cereals and others food products, may support the antioxidative defense [8,14,15,16].

Nevertheless, it is not only in the human body that oxidation damage may take place. Oxidation reactions are also present in many food products when exposed to air (oxygen) and/or to heat or light. In fact, food products’ deterioration processes are highly related to oxidation reactions and the decompositions of oxidation products. As such, antioxidants also play an important role in the maintenance of the products’ overall quality. One of the common deterioration processes is lipid peroxidation (e.g., in margarine, mayonnaise and frying oils) [17,18]. It causes the production of undesirable chemical compounds, such as aldehydes, ketones and organic acids, leading to the decrease of shelf life and nutritional value of lipid-containing food products [19]. The sensory impact of lipid oxidation is rancidity, responsible for changes in the flavor properties. In addition, the oxidative deterioration of lipids can lead to bleaching in foods due to the reaction with pigments, particularly carotenoids [20,21]. Enzymatic browning is another oxidative phenomenon during the maturation, processing and storage of food products. It involves the enzymatic oxidation of phenolic compounds, leading to the formation of dark pigments. Phenolic compounds act as substrates for oxidoreductases activities in the presence of oxygen; namely, polyphenoloxidases [22,23] and peroxidases [12,24,25]. These enzymes are the major contributors to changes in color, and eventually, to the final quality of many fruits and vegetables [26,27].

Since food products are not immediately consumed after their production, requiring storage and transportation, the suppliers have to ensure that they are delivered to consumers with safety and quality in mind, while possessing equal or higher nutritional value than when produced [28]. There is a constant search for strategies to increase food products’ overall qualities and shelf lives, in many cases by reducing or inhibiting oxidative damage; the incorporation of antioxidants is one of the strategies to delay the oxidation of biomolecules. Natural antioxidants, easily obtained from natural sources, possess great potential to be used as preservatives, replacing the synthetic ones [29].

Reviews on antioxidant sources have been published in the last decade. However, this paper is specially focused on the use of plant-source antioxidants as preservatives and packaging systems for food applications, including their sources, extractions and stabilization methods.

The research was performed using the databases PubMed, Google Scholar and ScienceDirect. The index terms employed for the search were in English, and the most recent articles pertinent to the theme were selected.

## 2. Replacing Synthetic Antioxidants with Natural Antioxidants

The choice of antioxidants for incorporation into food products is controlled by the regulatory laws of specific countries or by international standards. In the European Union, the regulation of antioxidants is established by the European Parliament and Council Directive No.1333/2008 of 16 December 2008 on food additives, which provides a list of approved additives and the conditions of their use and labelling.

Synthetic antioxidants have been used in place of natural ones, mainly because they present higher stability and performance, low costs and wide availability [19,30]. The most referenced synthetic antioxidants in the food industry are butylated hydroxyanisole (BHA), butylated hydroxytoluene (BHT), propyl gallate (PG) and tert-butyl hydroquinone (TBHQ). In addition, 2-naphthol (2NL), 4-phenylphenol (OPP) and 2,4-dichlorophenoxyacetic acid (2,4-DA) are the ones commonly used in fruits and vegetables [30].

Although synthetic antioxidants have been widely used, safety issues have been raised over time. There are several published studies indicating a relationship between the long-term intake of synthetic antioxidants and some health issues, such as skin allergies, gastrointestinal tract problems and in some cases increased the risk of cancer [31,32,33,34,35]. High doses of synthetic antioxidants may cause DNA damage and induce premature senescence [36]. BHA and BHT have already been found to be responsible for adverse effects on the liver and for carcinogenesis in animal studies [19,33]. Additionally, very little is known about the environmental occurrence and fate of these compounds [37,38]. The tendency to replace these antioxidants with natural ones has been increasing [39]. Studies related to the perception of consumers about the risks associated with using synthetic compounds for coloring and preserving food products have been performed. The conclusions have shown that consumers are concerned about being exposed in their daily diet to synthetic compounds, with a stronger preference for natural ones [40,41,42]. In addition, the use of natural antioxidants enables producers to satisfy the demands of consumers for cleaner-label products with exclusive natural ingredients. However, it should be emphasized that the fact of being from natural origin does not make them safe by default. There is still the need for toxicity studies for these compounds to define the conditions of their use in food products.

Natural antioxidants from plants may be classified into three main classes: phenolic compounds, vitamins and carotenoids [13,16,17]. Some phenolic compounds, in addition to being the major plant-compounds with antioxidant activity, also present antimicrobial and antifungal activities, and have important effects on the flavors and textures of food products [27]. Phenolic compounds show a large diversity of structures, from simple molecules (e.g., ferulic acid, vanillin, gallic acid and caffeic acid) to polyphenols like tannins and flavonoids [43]. Regarding vitamins, the most important include Vitamins E and C. The first is a lipid soluble vitamin consisting of a group of chemical compounds comprised of four tocopherols and four tocotrienols, which includes four isomers (α, β, γ and δ), but only α-tocopherol can be absorbed by the human body. It can be found mainly in legumes and cereal grains [44,45]. Vitamin C is soluble in water and is naturally present in many fruits and vegetables. Most carotenoids are also found in fruits and vegetables. β-carotene, α-carotene, lycopene and lutein are the main carotenoids with antioxidant activity [46]. In addition to their antioxidant capacities, they have the possibility to be used as food colorants [21].

There are many compounds able to inhibit oxidation, but only some of them are suitable for human consumption due to safety issues. Food grade antioxidants must be approved by regulatory bodies (generally recognized as safe (GRAS) level). They should not negatively affect color, odor or flavor; should be effective at low concentrations (0.001%–0.01%), should be compatible with the foods and have easy applications; should be stable during processing and storage; and should be economical. In addition, among other properties, antioxidants should have their LD_50_ values lower than 1000 mg/kg body weight, and should not have any significant effects on the growth of an experimental animal in long-term studies at a level 100 times greater than that proposed for human consumption [47]. Studies on their possible mutagenic, teratogenic and carcinogenic effects are also required [48]. Some natural antioxidants have lower antioxidant activities than their synthetic counterparts, which implies their use in larger amounts. This fact may lead to dosages that can be harmful [49]. Still, natural antioxidants are a valuable alternative approach to synthetic ones, providing they are used under the regulatory limits.

Beyond safety issues, the selection of natural extracts from plants is carried out taking into account the organoleptic characteristics of the food product, in order to avoid rejection by the consumers due to their characteristic colors or flavors [50]. The effect of the natural antioxidants (chamomile and fennel extracts) and synthetic ones (potassium sorbate) in the preservation of yogurt was carried out by Caleja and Barros [51]. The nutritional value of this food product did not have significant changes when adding either antioxidant. However, higher antioxidant activity was confirmed with the addition of natural ones, especially with chamomile decoction. Furthermore, the incorporation of fennel and chamomile extracts in biscuits showed that the antioxidant activity and the organoleptic and nutritional values were similar to the synthetic ones [52]. Some plant extracts used as antioxidants, such as from grape seeds, green tea, pine bark, rosemary, pomegranates and cinnamon, have also exhibited similar or better properties compared to synthetic antioxidants [53]. Estévez and Ventanas [54], reported that sage and rosemary essential oils exhibited similar antioxidant properties to BHT in refrigerated porcine liver pâté. This trend has been increasing the interest of researchers for new raw materials with antioxidant power (such as by-products from the agricultural-food industry), without affecting the consumers’ perceptions and the quality of the final products, and at the same time, producing a functional food with added value [29].

## 3. The Safety and Toxicity of Natural Antioxidants

Food additives are subjected to the same strict safety standards regardless of whether they are naturally or synthetically derived. The safety of food additives, in which natural antioxidants are included, is determined by considering potential cumulative effects that are evaluated by the outcome of toxicity studies and from knowledge about the chemical compounds [55]. There are several reports in the literature regarding the importance of safety assessments and toxicological tests carried out on specific natural extracts. As examples, acute and subchronic toxicological tests were performed for bamboo leaf extracts, which were generally regarded as safe by the authors for use as food additives [56]. In addition, the hydroethanolic extract of *Dolichandra unguis-cati* leaves did not present relevant toxic effects when administered orally to male and female rats under acute and subacute tests [57]. The subchronic toxicity and genotoxicity of the flavonoid-rich extract from *Maydis stigma* were examined. The results exonerate its safe use as a functional food, food additive and natural remedy [58]. Toxicological studies of essential oils with the antioxidant activity of autochthonous-flavoring herbs from Portugal revealed low toxicity in Swiss mice (DL_50_ > 1000 mg/kg) [59].

However, to use antioxidants in food products, they must undergo premarket approval by the European Food Safety Authority (EFSA) or by the United States Food and Drug Administration, (FDA), which have standard methodologies to assess their safety.

According EFSA, the scientific data required for the safety evaluation of a food additive follows four main levels: the chemistry and specifications of the substance (in terms of chemical structures and physicochemical properties); the existing authorizations and evaluations (an overview of previous risk assessments on the additive); the proposed uses and exposure assessment; and toxicological studies [60]. For the toxicological studies, there is a tiered approach consisting of three tiers (Table 1), where a minimal number of tests to all compounds should be carried out under tier 1, while tier 2 tests will be required for compounds which demonstrate absorption, toxicity or genotoxicity in tier 1 tests. Tier 3 tests should be performed on a case-by-case basis, to elucidate specific endpoints needing further investigation of the findings from tier 2 tests.

As an example, an extract of rosemary was authorized for use as a food additive in the European Union in several food categories, with a maximum level that has been identified as E 392 [61].

Concerning the FDA, premarket approval is based in a similar methodology to that of EFSA (Table 2). The additive concern level is estimated based on information on its toxicological potential predicted from its chemical structure, being one of three broad categories (A, B or C) according to an estimation of cumulative human exposure [55]. Category A structures with cumulative human exposure from 0 to 50 parts per billion (ppb, equivalent to microgram per kg diet) fall into Concern Level (CL) I; from 50 ppb to 1000 ppb lands in CL II; and above 1000 ppb fall into CL III.

Category B structures with cumulative human exposure from 0 to 25 ppb fall into CL I; from 25 ppb to 500 ppb are in CL II; and above 500 ppb fall into CL III. Furthermore, Category C structures with cumulative human exposure from 0 to 12 ppb fall into CL I; from 12 to 250 ppb lands in CL II; and above 250 ppb fall into CL III. After that, there are recommendations for the minimum toxicity tests to be performed for safety evaluations of food additives based on their levels of concern.

## 4. Natural Sources of Antioxidants

Most of the natural antioxidants are derived from plant materials, such as fruits, vegetables, herbs and spices [62,63,64]. These are particularly rich in phenolic compounds, vitamins and carotenoids [16,65,66]. Halvorsen and Holte [67], stated that *Rosaceae, Empetraceae, Ericaceae, Grossulariaceae, Juglandaceae, Asteraceae, Punicaceae* and *Zingiberaceae* are families of plants that contain compounds with high antioxidant activities, which include fruits, such as blackberries, strawberries, blueberries, black currants, walnuts, pomegranates and others. Essential oils from spices and herbs, such as oregano, thyme, dittany, marjoram, lavender and rosemary, have also been demonstrated to be excellent sources of natural antioxidant molecules, but with more limited ranges of applications due to their strong flavor characteristics [39,68]. Aqueous tea extracts have also been used as sources of natural antioxidants because of their contents of several compounds, such as catechins, tannins and other flavonoids, with the advantage of not presenting a strong flavor like essentials oils [69].

Concerning fruit and legumes, the processing industries are constantly struggling to reduce by-products, not only because the environmental problems associated, but due to the socio-economic losses [29]. For example, in fruit processing, as in the case of production of juices, pulps, canned fruit and others, industries generate particular by-products in the form of peels, cores, seeds, leaves and others that are discarded. More than half of the agricultural-food residues are derived from this sector, so a change of behavior, including initiatives and projects aimed at reducing the byproducts of processing, are highly attractive [65].

Along those lines, by-products of fruit and legume processing can be a source of functional compounds, such as apple pomace that has shown to be a good source of polyphenols, especially the peel [70]; grape pomace for its rich composition in anthocyanins, catechins, flavanols, phenolic acids and stilbenes [71]; tomato pomace rich in lycopene and other carotenoids [72]; and olive pomace rich in phenolic compounds [73]. Pomegranate is an example of a fruit extremely rich in antioxidants, mainly polyphenols, present in its edible and non-edible parts. According to the literature for each ton of pomegranate juice produced, nine tones of by-products are formed, which can be used as a natural source of bioactive compounds [74].

The valorization of by-products with the recovery of antioxidant rich extracts is even more interesting knowing the fact that the non-edible parts of fruits often contain a higher bioactive contents than the edible parts. Gorinstein and Martín-Belloso [75], found that the peels of some citrus fruits, such as lemons, oranges and grapefruit, presented phenolic compound contents 15% higher than the peeled fruits. Concerning pineapple by-products, their total phenolic content (13.79 mg of gallic acid equivalents 100 g^−1^) was higher when compared to that of fresh pulp (2.71 mg of gallic acid equivalents 100 g^−1^) [76]. Freitas and Moldão-Martins [65], also reported high contents of bioactive compounds—namely, carotenoids (β-carotene) and vitamin C, in pineapple rinds and cores—which impart high antioxidant potentials. The same trend was presented by George and Kaur [77], who evaluated the contents of different antioxidants, such as lycopene, ascorbic acid and phenolic compounds in tomatoes, and also noticed that peels had significantly higher antioxidant contents than the pulp.

It is important to emphasize that when dealing with plant sources it is essential to maintain as constantly as possible, several factors, such as the cultivar, climatic and soil conditions, harvest season, post-harvest conditions and extraction processes. These factors will have a strong impact on natural extracts’ standardizations.

## 5. Extraction Processes of Natural Antioxidants

As mentioned, many natural antioxidants are contained within vegetal matrices and their separation for further utilization is needed. Antioxidants can be extracted from different plant parts such as leaves, roots, stems, fruits, seeds and peels [53]. The quality of natural extracts and their antioxidant power depends not only on the quality of the original source (e.g., geographic origin, nutritional aspects and storage) but also on the technologies applied for their extraction.

So far, extraction processes have been mainly performed at laboratory-scales. Scale-up is not direct because it strongly depends, for example, on complex transport phenomena. However, some authors have already reported applicability at higher scales. Périno and Pierson [78], optimized the extraction of polyphenols from lettuce at a pilot scale by solvent-free microwave extraction. Saffarzadeh-Matin and Khosrowshahi [79], used solvent extraction for phenolic compounds from pomegranate waste, which was also successfully implemented at a pilot plant scale. In addition, Solana and Mirofci [80] studied the scaling-up of the supercritical fluid extraction of phenolic and glucosinolate from rocket salad.

There is not one standard procedure for performing the extraction of all natural antioxidants, since each compound has its own chemical and physical properties, and they are present in quite different solid matrixes. Still, extraction using organic solvents is the most common process used [81,82]. Along with conventional solvent extraction procedures, other strategies may be used, such as extraction with supercritical fluids, high hydrostatic pressure, microwaves and ultrasound [12]. However, these more recent technologies usually present higher investment costs, but often lower environmental impact [78]. Table 3 summarizes some examples of methods used for the extractions of natural antioxidant compounds from different sources.

The extraction yield and antioxidant capacity are dependent on the solvent, on the conditions under which the process is carried out and on the extraction method used. Those factors may affect the amounts and qualities of antioxidants in the extracts; for example, due to breakdown and polymerization reactions [123]. An efficient extraction is obtained when it is possible to extract the maximum amounts of the bioactive compounds with the lowest degradation degree of the compounds, and minimum amounts of non-antioxidant substances, such as sugars and organic acids [124].

### 5.1. Conventional Extraction Techniques

Solvent extraction (solid–liquid or liquid–liquid) involves the choice of solvents and the use of heat and/or stirring [125]. A solid–liquid extraction process is normally executed in a Soxhlet apparatus, where the plant material is placed together with a condensed solvent. The advantages of using Soxhlet include, among others, the repetitiveness of placing fresh solvent in contact with the solid matrix, and that no filtration is required at the end of the process. However, it has disadvantages, such as the need for large quantities of solvents, and consequently, an evaporation/concentration process; not having stirring during the process; and the possible thermal degradation of the compounds, as the process is usually carried out at the boiling point of the solvents for a long period of time [63,82,126].

When dealing with plant materials rich in a wide range of phenolic compounds, the extraction yield depends on various factors, such as the type of the solvent used (polarity), extraction temperature, time and solvent-to-plant ratio. The choice of the solvent depends on the nature of the compounds that are intended to be extracted, being that the extraction yield is influenced by their solubilities in the solvent to be used. Solvent-to-plant ratio should be optimized in order to use a suitable solvent concentration that prevents its saturation in molecules extracted during the extraction process [87,89]. Since they may be attached to insoluble components, such as waxes, terpenes or fats, a preliminary solid-liquid extraction process may be required for the removal of the unwanted phenolics and non-phenolic substances [81].

Different solvents have been used, separately or in mixtures, including ethanol [77], acetone [42], methanol [88], hexane [70] and water [94]. In the study of Peschel and Sánchez-Rabaneda [29], fruit and vegetable by-products (from red beet, apple, strawberry, pear, artichoke, asparagus, tomato, broccoli, cucumber, endive, chicory, golden rod and woad herb) were extracted with five solvents: water, methanol, ethanol, acetone and hexane. It was found that, in general, a higher yield resulted from a one-step extraction using water and methanol compared to the other methods. In contrast, the extraction of anthocyanins and polyphenols from grapes and red and black currants was more efficient using ethanol and methanol compared to water [127]. Additionally, Boulekbache-Makhlouf and Medouni [88], studied different solvents to quantify the total phenolics, flavonoids, tannins and anthocyanins in eggplant peel. They concluded that methanol was the best solvent for the extraction of anthocyanins, mainly because they are polar molecules, and acetone was described as the best solvent for quantifying the other compounds. Fu and Tu [85] studied phenolics and anthocyanin extraction from sweet potato leaves with three solvents (water, ethanol and acetone). They reported that the extraction with 70% ethanol resulted in extracts with the highest total flavonoid and total anthocyanin contents. Though, 50% acetone was the solvent producing extracts with higher total phenolic content.

Although there are a large number of works in the literature focused on extraction yield, it is not clear which solvent is more effective for a specific raw material. From the food industry point of view, from all solvents mentioned, ethanol and water are the more adequate, as they have GRAS (generally recognized as safe) status [12,124].

### 5.2. Non-Conventional Extraction Techniques

#### 5.2.1. Supercritical Fluid Extraction (SFE)

SFE is an environmentally-friendly, alternative process to conventional organic solvent extraction, as it uses as solvents fluids in their supercritical states, avoiding the use of large quantities of toxic solvents that are undesirable in the food industry. In addition to this, it is carried out in the absence of light and oxygen, which reduces the degradation of the compounds [35,128]. Carbon dioxide is the most commonly-used solvent to obtain supercritical conditions, mainly due to its low toxicity, low cost, easy separation from extracted solutes, compatibility with foodstuffs and possibility to be used when low temperatures are required [129]. SFE with carbon dioxide is an efficient alternative process to conventional solvent extraction methods, especially for extracting lipophilic plant materials, such as lipids, essential oils and aroma compounds. The same does not happen with more hydrophilic substances, such as phenolics, alkaloids and glycosidic compounds, which are poorly soluble in supercritical carbon dioxide [126]. This process was applied, for example, in the extractions of carotenoids from mango peel [98] and phenolic compounds from apple pomace [99].

#### 5.2.2. High Hydrostatic Pressure (HHP) and Pressurized Liquid Extraction (PLE)

In both HPP and PLE processes, the pressure is applied in order to increase mass transfer rate between solid matrices and extraction solvent, resulting in fast and effective alternative methods for active compounds’ extractions. However, high pressure can enhance the extraction efficiency, but with higher investment costs.

When applying HHP, a raw material/solvent mixture is hermetically sealed in a package and introduced to a vessel containing a pressure-transmitting medium (e.g., water, hydrophilic and lipophilic organic solvents at different concentrations) [86,104]. Pressure is applied, normally in the range of 100–1000 MPa, at a controlled temperature, including ambient temperature, which is an advantage when heat sensitive compounds are involved [106]. This method has been used successfully, for example, in the extraction of flavonoids and lycopene from tomato pulp [104] and phenolic acids and flavonoids from watercress [105]. In addition, Briones-Labarca and Plaza-Morales [106], demonstrated that HHP enabled a higher yield and shorter extraction time, compared to other extraction methods (ultrasounds and conventional extractions) when applied to recover phenolic compounds and oil from papaya seeds.

In the case of the PLE process, the material/solvent mixture is directly introduced in a closed vessel, after which high pressures (3.3−20.3 MPa) and temperatures (40−200 °C) are applied, enabling rapid extraction periods (3−20 min). Pressurized solvents remain in liquid states above their boiling points, allowing for high-temperature extraction. There is an improvement of compounds’ solubilities and the desorption kinetics from the matrices [130]. This process has been used to extract several bioactive natural compounds, such as essential oil from peppermint [107], polyphenols from goldenberry [109], carotenoids from carrot by-products [108] and phenolic compounds from a large variety of plant materials (e.g., Goji berry, feijoa peel, grape marc, spent coffee grinds and olive pomace) [25,110,111,112,113].

#### 5.2.3. Ultrasound-Assisted Extraction (UAE)

UAE is a method in which ultrasound is applied to a mixture of a solvents with the solids containing the target molecules. It is a process whereby a combination of mixing effects with physical impacts of ultrasound on raw material (e.g., fragmentation, erosion, sonocapillary effect, sonoporation, local shear stress and disintegration of plant cell walls) increases the mass transfer rate, which may explain its enhanced extraction performance. Eventually, it allows one to reduce the time of extraction, the amount of solvent used and the energy consumption. In addition, ultrasound may also be applied in combination with other methods, such as soxhlet extraction, microwave extraction, supercritical fluid extraction and extrusion [131]. UAE has been used in the extraction of bioactive compounds, such as carotenoids from pomegranate wastes [116], phenolics from blueberry pomace [117] and edible oils from plant sources [126,132]. This technology has also been used as a supplementary technique to conventional ones. Guandalini and Rodrigues [118] used ultrasound for pectin recovery, after the extraction of phenolic compounds from mango peel with a conventional ethanol–water extraction, with the purpose of generating value from the whole residue obtained.

#### 5.2.4. Microwave-Assisted Extraction (MAE)

Using microwaves is another green extraction method, which is based on the direct impact on polar compounds. MAE offers rapid delivery of energy to a total volume of solvent and solid matrix with subsequent efficient and homogenous heating of both phases. It enables the reduction of the extraction time and solvent volumes, and can be performed in open or closed systems. In the latter case, the solvent and sample are contained in sealed vessels under a controlled temperature and pressure. The closed vessels allow the temperature of the solvent to rise above its boiling point, which decreases extraction time and subsequently increases extraction efficiency [133]. However, when solvents are non-polar or volatile, the efficiency of this method can be very low. As such, polar solvents, such as ethanol, methanol and water should be used [126]. This process has been applied to recover antioxidants from a large number of plant materials, such as phenolics from pomegranate peels [119] and carotenoids from Gac peel [121]. Even though MAE is commonly applied to solid/solvent mixtures, works have been reported in which microwaves were applied only to the plant material; namely, in the extraction of polyphenols from olive tree leaves [122].

## 6. Stabilization Processes

After extraction, natural antioxidants are susceptible to degradation during storage and when incorporated in food products, since they present a high sensitivity to environmental conditions (e.g., temperature, pH, light, oxygen and moisture). In order to increase their stability and avoid nutritional and functional losses, the degradation rate should be minimized. There is not one universal technique for performing antioxidant stabilization, since each compound/natural extract has specific physical and chemical characteristics. Ideally, the method should be simple, fast and easy to reproduce at an industrial scale at low cost. The most common techniques can be divided into chemical process (e.g., coacervation or molecular inclusion in cyclodextrins), and mechanical processes (e.g., spray drying, fluid bed coating, spray cooling/chilling, extrusion, emulsification and freeze drying) [134,135].

Stabilization is generally achieved with the production of capsule structures (from nano to micrometer range), which are composed of a core and a wall material. The core of capsules consists of the ingredients to be protected, and the wall material—also denominated as the coating, shell, membrane, carrier or coat—is the external layer or layers that cover the core, made of food grade materials (e.g., polysaccharides, proteins and lipids).

Beyond the protection of the bioactives against degradation reactions promoted by external factors, the encapsulation in the form of micro/nanoparticles for the food industry also enables easier handling of bioactive compounds that may be in another physical state (e.g., converting a liquid active compound into a powder); the prevention of evaporation and degradation of volatile active compounds, such as essential oils, masking negative organoleptic properties such as color, flavor and odor of the natural antioxidants; the controlled release of the encapsulated materials at a specific time and place in the human body; and their immobilization in the food processing systems [135,136,137].

The most common process used is spray drying, due to its low cost, easy scale-up and applicability with a wide range of core and wall materials, delivering the encapsulated materials as a powder with particles generally a few micrometers in size. It has been used, for example, to encapsulate phenolic compounds from spent espresso coffee [110]; olive leaf extracts [138]; citrus by-product extracts [139]; apple fruit peel [140]; pigments from wine by-products [141]; carotenoids from tomato pomace [72]; essential oils from herbs, such as oregano [142,143,144], and lavender [145]; and vitamins [146].

## 7. Applications in the Food Industry

Natural antioxidants have been studied in a wide range of applications, like as preservatives in several food products [147], in edible coatings [148] and in films to be used in food packaging [149].

### 7.1. Plant Extracts and Essential Oils as Natural Antioxidants

Several works have been published with the purpose of studying the incorporation of natural antioxidants into food matrices. Some of them are presented in Table 4.

Phenolic compounds are the main class of antioxidants used in food industry. Furthermore, many of these natural ingredients also present the capacity to delay or inhibit the growth of pathogenic microorganisms in food, such as *Salmonella* spp. and *Escherichia Coli* [162].

Among the studies carried out in recent years, much attention was paid to the use of natural antioxidants in meat and meat products, including the regulatory aspects concerning the replacement of synthetic antioxidants by natural ingredients [163].

Meat fat presents a high susceptibility to oxidation, especially the unsaturated fatty acid fractions, which are generally higher in poultry meat compared to cattle and pig meats. Many of the oxidation products are responsible for rancidity, decreased nutrient value and increased health risk, due to accumulation of toxic compounds. The incorporation of natural antioxidants can reduce or minimize the formation of chemical toxins, increasing the nutritional status and health benefits of these products, as well as their shelf life. Balzan and Taticchi [164] used purified phenols from olive oil wastewater on raw and cooked sausages, which induced a strong decrease of several oxidation markers, maintaining the overall sensory acceptance. Das and Rajkumar [151], applied a litchi fruit pericarp extract in cooked nuggets of sheep meat during 12 days of refrigerated storage, which revealed itself to be effective at retarding oxidation by 1.5% without affecting the products’ acceptability. Jayathilakan and Sharma [152], reported effective protection of cooked meat’s quality when applying extracts from plants, such as cloves and cinnamon, to precooked mutton, beef and pork. These natural antioxidants did not introduce undesirable flavor attributes and prevented oxidative rancidity. Carotenoids have also been tested. The protective activity of tomato pomace extracts applied on the surface of lamb steaks packaged under a modified atmosphere improved their shelf life [153]. Through combinations with other compounds, natural antioxidants can provide advantages to the food product to which they are added. Kanatt and Chander [159], developed a mixture of chitosan and mint extracts, which together imparted antioxidant and antimicrobial properties, extending the shelf lives of meat and meat products.

Besides meat products, natural antioxidants have also been applied to enhance the stability of edible oils that do not contain natural antioxidants. The phenolic compounds from plant sources, such as spices, herbs, teas, oils, seeds, cereals, cocoa shell, fruits and vegetables, as natural antioxidants, were often used in a number of edible oils. Among the herbs, rosemary and oregano are the most studied sources of antioxidants in lipid systems. In addition, Özcan and Arslan [165] studied the antioxidant effects of essential oils from rosemary, clove and cinnamon on hazelnut and poppy oils. Among the essential oils investigated, the cinnamon oil was the most effective antioxidant at preventing lipid oxidation, which was followed by clove and rosemary essential oils. More recently, Bodoira and Penci [156] reported that commercial natural antioxidants (natural mixed tocopherols, rosemary extract, ascorbyl palmitate and citric acid) could be effective to prevent chia oil oxidation, and Galanakis and Tsatalas [23] have shown that polyphenols from olive mill wastewater are effective as preservatives in olive oils and refined olive kernel oils.

Natural antioxidants were also studied in bakery products. For example, Caleja and Barros [52] tested the incorporation of fennel and chamomile aqueous extracts in biscuits, showing strong antioxidant activity. These extracts did not cause significant changes in the appearances or nutritional profiles of the products; thus, implying that the use of such extracts could be important for the release of new, healthier pastry products on the market.

Most of the published studies on the use of natural antioxidants have shown great results in the inhibition of and control over the oxidative process. However, the amount of bioactive compounds added is an important factor in their efficacy, which is restricted, not only by legislation, but also by sensory acceptance. Rejection by consumers occurs mainly when natural antioxidants are in the form of plant essential oils (e.g., from oregano, rosemary or lavender), which are volatile and impart a strong flavor. Encapsulation processes (e.g., by spray drying) or their incorporation in coatings and films have been proposed to mask the strong flavor.

Color is one of the most important properties for the acceptance of food products by consumers. Natural antioxidants may be used in the prevention of color changes due to pigments’ oxidation in food systems. In fresh fruit juices, natural antioxidants have been proposed for improving the stability of carotenoid pigments, as well as for aroma protection and stabilization [166]. Caleja and Barros [28] demonstrated that the incorporation of fennel and chamomile extracts into cottage cheese allows it to avoid changing in color (yellowness) during storage, which was confirmed with a control sample. Moreover, the stabilization by microencapsulation has been studied for improving the antioxidant activity for longer storage times [167].

In the case of meat products, the color is also a strong indicator of quality, pertaining to things such as meat oxidation. Myoglobin is a heme protein able to store oxygen in muscle tissues, which is responsible for meat pigmentation, exhibiting different redox states depending on the state of the chelated Fe ion. In contact with oxygen, it confers the oxidation of heme iron from the ferrous state in oxymyoglobin (oxygenated myoglobin) to the ferric state in metmyoglobin, causing a brownish-red color. This oxidation reaction can be delayed by the addition of antioxidants [168]. Vitamin E allows one control the color deterioration by decreasing the rate of myoglobin oxidation in beef [169]. Moreover, Fernández-López and Sevilla [161], investigated pigment degradation caused by the cooking and storage of pork meat. These authors found that the addition of rosemary and hyssop extracts stabilized the meat color and consequently increased the shelf life of the products. Mansour and Khalil [50], in their study with freeze-dried extracts of ginger rhizomes and fenugreek seeds added to beef patties, found them to be active in retarding rancid odor and color change. Furthermore, those extracts exhibited higher antioxidant activities compared to commercial antioxidants during cold storage. In another study by Karre and Lopez [157], it was reported that the antioxidant potentials of fruits, such as plums, grape seeds, cranberries, pomegranates and bearberries; and plants, such as rosemary, oregano and pine bark, for meat and poultry products, positively affected product qualities. However, when using extracts rich in phenolic compounds, some potently active polyphenols may accelerate the oxidation of oxymyoglobin due to the pro-oxidant nature of their quinone derivatives [168]. Lycopene extract from a tomato source was added to minced meat, increasing its storage stability by reducing the microbiological activity. In addition, it improved color and added a natural taste. This extract exerted several functionalities at the same time (pigment, antioxidant and antimicrobial activity), presenting great potential for use in meat products. However, studies regarding adverse effects, like pro-oxidant reactions, are still necessary [170].

### 7.2. Antioxidant Edible Coatings and Films

Alternatively to the incorporation of natural antioxidants into food formulations, they have been used to produce protective active barriers to be applied directly in food products surface (edible coatings) or in films for food packaging purposes. These barriers represent a new approach to solving the detrimental impacts of oxygen on food [171]. Most of the natural antioxidants reported in the literature also possess antimicrobial activity. As such, these active barriers may be designed to extend food products’ shelf lives, as they are effective against not only oxidation reactions, such as enzymatic browning or oxidative rancidity, but against microbial proliferation. Edible films and coatings present, as main advantages over the antimicrobial or antioxidant agents, direct incorporation into bulk food, the possibility of active compounds’ diffusion control at the surface of the food and the reduction of the amount of preservatives added in the food. The production of these active barriers with natural biopolymers (e.g., chitosan, starch, alginate, whey protein and gelatin) has received increasing attention as an alternative to synthetic, non-biodegradable plastic packaging. A summary of recent works regarding active edible films and their applications for food preservation is presented in Table 5. 

Extracts from agricultural by-products have shown great potential to be incorporated in packaging materials, with promising results. Baranauskaite and Kopustinskiene [143], prepared chitosan films with an ethanolic mango leaf extract (MLE) having antioxidant properties. When applied as packaging materials for cashew nuts’ storage, fatty acid oxidation was reduced by 56% for the nuts stored with 5% MLE film compared to commercial, non-biodegradable polyamide/polyethylene (PA/PE) films. In a similar way, de Moraes Crizel and de Oliveira Rios [173], developed biodegradable films with antioxidant activities based on chitosan and olive pomace flour and microparticles, which showed better protective effects than PA/PE films, against the oxidation of walnuts stored under accelerated deterioration conditions for 31 days (T = 55 °C, with the incidence of UV fluorescence and 35% relative humidity). The same authors also reported the production and characterization of gelatin films with papaya peel microparticles [174]. The applications of these films as packaging materials of lard (used as food simulant) have shown a higher decrease of fat oxidation compared to commercial polyethylene. This effect was shown by the lower contents of primary and secondary products of oxidation quantified in lard packaged with the antioxidant films.

Qin and Zhang [186] produced chitosan and montmorillonite (MMT) active films enriched with pomegranate-rind ethanol extract presenting DPPH radical scavenging activity that was not affected by the addition of MMT. Other recent works have reported the developments of films with antioxidant activities, such as films made of arrowroot starch and blackberry particles [177]; fish gelatin or soy protein isolate with mango kernel ethanolic extract [176]; chitosan and Herba Lophatheri extract [178]; and chitosan or starch, containing aqueous thyme extract [175].

The addition of extracts to the films affects not only their antioxidant activity, but their other films properties. Martins and Arantes [187] developed gelatin films with commercial ginger gingko leaf and green tea extracts. Beyond higher antioxidant activity, the incorporation of those extracts improved their barrier properties against UV light and water vapor, respectively. In addition, a reduction of stress and strain at break of films with extracts was also observed compared to control films. In a similar way, carvacrol and pomegranate peel extracts also increased the barrier to water vapor transfer of chitosan films [188].

Coating the vegetables and fruits with edible materials has been used to reduce water vapor and gas exchanges, with the aim of increasing products’ shelf lives, and helping to maintain their firmness levels, colors, sensorial proprieties and antioxidant activities. These coatings have been used as carriers of bioactive compounds; namely, with antioxidant and antimicrobial properties. Recent works include Arabic edible gum coatings with tulsi extract rich in polyphenols and flavonoids, which was effective in the delay of guava fruits’ ripening rate. Furthermore, guava coated with the optimized formulation registered no mold growth [184]. In addition, guar gum and ginseng extract coating applied on sweet cherries controlled weight loss and reduced loss of ascorbic acid, polyphenols, titratable acidity and firmness compared to the control fruits. Moreover, the addition of ginseng extract decreased the polyphenol oxidase activity [179].

## 8. Conclusions

Antioxidants from natural sources are valuable bioactive compounds with well-demonstrated potentials for use in the food industry. Beyond their application in functional food products, attention has also been focused on their use as alternatives to their synthetic counterparts to increase product stability and avoid deterioration by oxidation during processing and storage. In the context of a circular economy, efforts are being dedicated to the use of natural antioxidants from food byproducts generated by the agricultural industry and from underexploited plant materials.

Each step between the extraction and the application of natural antioxidants has already been a focus of research. Regarding the extraction step, the selection of the most appropriate techniques differs according to the type of compounds targeted for recovery. More environmentally friendly techniques have been explored to avoid the large amounts of solvents used in conventional solvent extraction processes. Although replacing conventional technologies by non-conventional ones has emerged, improvements are necessary in terms of scaling up. Concerning the stabilization processes after extraction, spray drying has been the most-used process, mainly due to its simple operation and scaling up, delivering encapsulated antioxidants in the form of powder microparticles, enabling easy manipulation and dosages.

Although these compounds are derived from natural sources, their applications to food products must take into account their dosages and possible toxicological effects. Moreover, negative effects on sensory attributes, especially flavor and taste, imparted by some natural compounds, have to be addressed. This will increase the consumer propensity to purchase food products containing natural antioxidants, ultimately contributing to decreasing the prices of these products.

## Figures and Tables

**Table 1:** Toxicity tests requested by the European Food Safety Authority for safety evaluations of food additives.

Toxicity Tests		Tier 1	Tier 2	Tier 3
Toxicokinetics	Absorption	x	x	x
	ADME (single dose)		x	x
	ADME (repeated dose, volunteer studies)			x
Genotoxicity	in vitro testing	x	x	x
	in vivo testing		x	x
Toxicity	Extended 90-day toxicity study	x	x	x
	Chronic toxicity or Combined chronic		x	x
	Carcinogenicity or Combined		x	x
Reproductive and Developmental toxicity	EOGRTS		x	x
	Prenatal development toxicity		x	x
	Specialized studies (e.g., immunotoxicity, neurotoxicity, endocrine activity, mode of action)			x

**Table 2:** Toxicity tests requested by the US Food and Drug Administration for safety evaluations of food additives.

Toxicity Tests	CL Low (I)	CL Intermediate (II)	CL High (III)
Genetic toxicity tests	x	x	x
Short-term toxicity tests with rodents	x	x	x
Subchronic toxicity studies with rodents		x	x
Subchronic toxicity studies with non-rodents		x	x
One-year toxicity studies with non-rodents			x
Chronic toxicity or Combined chronic toxicity/carcinogenicity studies with rodents			x
Carcinogenicity studies with rodents			x
Reproduction studies		x	x
Developmental toxicity studies		x	x
Metabolism and Pharmacokinetic studies		x	x
Human studies			x

**Table 3:** Methods for antioxidants’ extraction from natural sources.

Extraction Process	Source	Antioxidant Extracted	References
*Organic Solvents:*			
Ethanol, dichloromethane, hexane	Coffee leaves	Chlorophylls and carotenoids	Marcheafave, Tormena [83]
Ethanol, acetone and water	*Baccharides* specie	Phenolic content	Casagrande, Zanela [84]
	Sweet potato	Polyphenols and anthocyanins	Fu, Tu [85]
Methanol, ethanol and acetone	Spent grain	Phenolic content	Socaci, Fărcaş [86]
	Spice herb	Phenolic content	Do, Angkawijaya [87]
	Peel of eggplant	Total phenolics, flavonoids, tannins and anthocyanins	Boulekbache-Makhlouf, Medouni [88]
Methanol, ethanol, acetone and water	Peach fruit	Flavonoids and phenolic compounds	Mokrani and Madani [89]
Ethanol	Jussara fruit	Phenolic compounds	de O. Silva, N. Castelo-Branco [90]
Acetone, acetic acid, water	Basil leaves	Phenolic compounds	Złotek, Mikulska [91]
Water, acetone and ethanol	Ginkgo biloba leaves	Flavonols	Kobus-Cisowska, Flaczyk [42]
N-hexane and methanol	Pineapple peel	Polyphenols	Li, Shen [92]
Hexane, ethyl acetate, chloroform, butanol, methanol and water	Ripe bananas	Phenols and flavonoids	Amri and Hossain [93]
Methanol, water and ethanol	Chestnut byproducts	Polyphenols	Vella, Laratta [94]
	Kumquat peel	Phenolic and flavonoid content	Lou, Lai [95]
Water and ethanol	*Withnaia somnifera* herb	Phenolic compounds	Dhanani, Shah [96]
	Propolis	Phenolic compounds	Sun, Wu [97]
*Supercritical fluid extraction (SFE)*	Mango peel	Carotenoids	Sánchez-Camargo, Gutiérrez [98]
	Apple pomace	Phenolic compounds	Ferrentino, Morozova [99]
	Myrtle leaves and berries	Phenolic acids, flavonoids and anthocyanins	Pereira, Cebola [100]
	Green algae	Carotenoids and phenolic compounds	Fabrowska, Ibañez [101]
	Cape gooseberry	Phenolic compounds and β-carotene	Torres-Ossandón, Vega-Gálvez [102]
*High Hydrostatic Pressure (HHP)*	Red macroalgae	Proteins, polyphenols and polysaccharides	Suwal, Perreault [103]
	Tomato pulp	Flavonoids and lycopene	Briones-Labarca, Giovagnoli-Vicuña [104]
	Watercress	Phenolic acids and flavonoids from watercress	Pinela, Prieto [105]
	Papaya seeds	Phenolic content	Briones-Labarca, Plaza-Morales [106]
*Pressurized liquid extraction (PLE)*	Peppermint	Phenolic compounds and essential oils	Çam, Yüksel [107]
	Carrot by-products	Carotenoids	Mustafa [108]
	Goldenberry	Polyphenols	Corazza, Bilibio [109]
	Goji berry	Phenolic compounds	Tripodo, Ibáñez [25]
	Feijoa peel	Phenolic compounds	Abrahão, Rocha [110]
	Grape marc	Anthocyanins and phenolic compounds	Pereira, Tarone [111]
	Spent coffee ground	Polyphenols	Mariotti-Celis, Martínez-Cifuentes [112]
	Olive pomace	Phenolic compounds	Cea Pavez [113]
*Ultrasound-assisted extraction*	Green propolis	Phenolic compounds	Cavalaro, da Cruz [114]
	*Strawberry trees*	Anthocyanins	López, Caleja [115]
	Carotenoids	Pomegranate wastes	Goula, Ververi [116]
	Blueberry pomace	Phenolic compounds	Shi, Tranchant [117]
	Mango peel	Pectin and phenolic compounds	Guandalini, Rodrigues [118]
*Microwave-assisted extraction (MAE)*	Pomegranate peels	Phenolic compounds	Kaderides, Papaoikonomou [119]
	*Phaleria macrocarpa* fruit peel	Phenolic compounds	Alara, Mudalip [120]
	Gac peel	Carotenoids	Chuyen, Nguyen [121]
	Olive tree leaves	Phenolic compounds	Şahin, Samli [122]

**Table 4:** Applications of natural antioxidants in food products.

Natural Source	Main Active Compound	Food Matrix	Reference
Fennel and chamomile aqueous extracts	Phenolic compounds	Biscuits	Caleja, Barros [52]
		Cottage-cheese	Caleja, Barros [147] Caleja, Barros [28]
		Yogurt	Caleja, Barros [51]
Olive leaf and cakes extracts by-products	Phenolic compounds	Antioxidant film	Moudache, Colon [150]
Litchi fruit pericarp extract	Phenolic compounds	Cooked nuggets	Das, Rajkumar [151]
Green tea extract	Polyphenols	Sunflower oil	Yin, Becker [69]
Cloves and cinnamon	Phenolic compounds	Meat samples	Jayathilakan, Sharma [152]
Tomato pomace extract	Carotenoids	Lamb steaks packaged	Andres, Petron [153]
*Ginkgo biloba* leaves extract	Polyphenols	Pork meat	Kobus-Cisowska, Flaczyk [42]
Cloudberry, beetroot and willow herb	Flavonoid	Cooked pork patties	Rey, Hopia [154]
Canola olive oils, rice bran and walnut	Polyphenols, vitamins E and B	Pork frankfurters	Álvarez, Xiong [155]
Rosemary extract	Phenolic compounds	chia oil oxidation	Bodoira, Penci [156]
Olive mill wastewater	Polyphenols	Olive oils and refined olive kernel oils	Galanakis, Tsatalas [23]
Potato peels, fenugreek seed and ginger rhizomes	Phenolic compounds	Ground beef patties	Mansour and Khalil [50]
Olive mill wastemater	Polyphenols, ascorbic acid and tocopherols	Bread and rusks	Galanakis, Tsatalas [22]
Fruits and plants	Phenolic compounds	Meat and poultry products	Karre, Lopez [157]
Apple peel	Phenolic compounds	Tomato juice	Massini, Rico [158]
Chitosan and Mint extract	Phenolic compounds	Pork cocktail Salami	Kanatt, Chander [159]
Extracts from the hard winter wheat	Phenolic compounds	Fish oils	Yu, Haley [160]
Rosemary and hyssop extracts	Phenolic compounds	Pork meat	Fernández-López, Sevilla [161]

**Table 5:** Application of antioxidant-enriched edible films and edible coatings for food preservation.

	Source of Antioxidants	Main Active Compound	Biopolymers Matrix	References
**Edible films**	Propolis extract	Flavonoid aglycones, phenolic acids and their esters, phenolic aldehydes, alcohols, ketones	Chitosan	Siripatrawan and Vitchayakitti [172]
	Olive pomace	Phenolic compounds and carotenoids	Chitosan	de Moraes Crizel, de Oliveira Rios [173]
	Papaya peel microparticles	Phenolics and carotenoids	Gelatin	de Moraes Crizel, de Oliveira Rios [174]
	Thyme extract	Caffeic acid, flavonoid glycosides, hydroquinones derivates, terpenoids and biphenyl compounds	Chitosan and starch	Talón, Trifkovic [175]
	Mango kernel extract	Gallic acid, ellagic acid, ferulic acid, cinnamic acids, tannins, vanillin, coumarin and mangiferin	Soy protein isolate and fish gelatin	Maryam Adilah, Jamilah [176]
	Mango leaf extract	Gallic acid, mangiferin, glucosides and other phenolic compounds	Chitosan	Baranauskaite, Kopustinskiene [143]
	Blackberry powder	Phenolic acids, tannins and anthocyanins	Arrowroot starch films	Nogueira, Fakhouri [177]
	*Herba Lophatheri* extract	Flavonoids	Chitosan	Wang, Guo [178]
**Edible coatings**	Oregano essential oil	Thymol, ρ-cymene and carvacrol	Pectin	Rodriguez-Garcia, Cruz-Valenzuela [179]
	Ginger essential oil	α-zingiberene and β-sesquiphellandrene	Sodium caseinate	Noori, Zeynali [180]
	Roselle calyces extract and cinnamon essential oil	Anthocyanins	Chitosan	Ventura-Aguilar, Bautista-Baños [181]
	Rosemary extract and essential oil	Rosmarinic acid, caffeic acid, flavonoids, 1,8-cineole, L-camphor, a-pinene and 1-borneol	Carboxyl methyl cellulose	Choulitoudi, Ganiari [182]
	Essential oil and extract from *Satureja thymbra*	Υ-Terpinene and carvacrol	Carboxyl methyl cellulose	Choulitoudi, Bravou [183]
	Tulsi extract	Polyphenols and flavonoids	Arabic gum	Murmu and Mishra [184]
	Ginseng extract	Ginsenosides, alkaloids, polysaccharides, phytosterols, polyacetylenes, phenolics and limonene	Guar gum	Dong and Wang [185]

## References

[1] Lü J.M., Lin P.H., Yao Q., Chen C. (2010). Chemical and molecular mechanisms of antioxidants: Experimental approaches and model systems. J. Cell. Mol. Med..

[2] Carocho M., Ferreira I.C. (2013). A review on antioxidants, prooxidants and related controversy: Natural and synthetic compounds, screening and analysis methodologies and future perspectives. Food Chem. Toxicol..

[3] Lobo V., Patil A., Phatak A., Chandra N. (2010). Free radicals, antioxidants and functional foods: Impact on human health. Pharmacogn. Rev..

[4] Augustyniak A., Bartosz G., Čipak A., Duburs G., Horáková L., Łuczaj W., Majekova M., Odysseos A.D., Račková L., Skrzydlewska E. (2010). Natural and synthetic antioxidants: An updated overview. Free. Radic. Res..

[5] Valko M., Leibfritz D., Moncol J., Cronin M.T., Mazúr M., Telser J. (2007). Free radicals and antioxidants in normal physiological functions and human disease. Int. J. Biochem. Cell Boil..

[6] Valko M., Rhodes C., Moncol J., Izakovic M., Mazur M. (2006). Free radicals, metals and antioxidants in oxidative stress-induced cancer. Chem. Interact..

[7] Rajendran P., Nandakumar N., Rengarajan T., Palaniswami R., Gnanadhas E.N., Lakshminarasaiah U., Gopas J., Nishigaki I. (2014). Antioxidants and human diseases. Clin. Chim. Acta.

[8] Valavanidis A., Vlachogianni T., Fiotakis K., Loridas S. (2013). Pulmonary Oxidative Stress, Inflammation and Cancer: Respirable Particulate Matter, Fibrous Dusts and Ozone as Major Causes of Lung Carcinogenesis through Reactive Oxygen Species Mechanisms. Int. J. Environ. Res. Public Heal..

[9] Hadjigogos K. (2003). The role of free radicals in the pathogenesis of rheumatoid arthritis. Panminerva Med..

[10] Wojtunik-Kulesza K.A., Oniszczuk A., Oniszczuk T., Waksmundzka-Hajnos M. (2016). The influence of common free radicals and antioxidants on development of Alzheimer’s Disease. Biomed. Pharmacother..

[11] Getoff N. (2007). Anti-aging and aging factors in life. The role of free radicals. Radiat. Phys. Chem..

[12] Oroian M., Escriche I. (2015). Antioxidants: Characterization, natural sources, extraction and analysis. Food Res. Int..

[13] Dorman H., Peltoketo A., Hiltunen R., Tikkanen M. (2003). Characterisation of the antioxidant properties of de-odourised aqueous extracts from selected Lamiaceae herbs. Food Chem..

[14] Zunino S.J., Storms D.H., Stephensen C.B. (2007). Diets Rich in Polyphenols and Vitamin A Inhibit the Development of Type I Autoimmune Diabetes in Nonobese Diabetic Mice1-3. J. Nutr..

[15] Möller P., Loft S. (2006). Dietary antioxidants and beneficial effect on oxidatively damaged DNA. Free. Radic. Boil. Med..

[16] Sikora E., Cieślik E., Topolska K. (2008). The sources of natural antioxidants. Acta Sci. Pol. Technol. Aliment..

[17] Jayaprakasha G., Singh R., Sakariah K. (2001). Antioxidant activity of grape seed (Vitis vinifera) extracts on peroxidation models in vitro. Food Chem..

[18] Carocho M., Barreiro M.F., Morales P., Ferreira I.C., Gomez P.M. (2014). Adding Molecules to Food, Pros and Cons: A Review on Synthetic and Natural Food Additives. Compr. Rev. Food Sci. Food Saf..

[19] Saad B., Sing Y.Y., Nawi M.A., Hashim N., Mohamedali A., Saleh M.I., Sulaiman S.F., Talib K., Ahmad K., Ali A.S.M. (2007). Determination of synthetic phenolic antioxidants in food items using reversed-phase HPLC. Food Chem..

[20] Li J., Solval K.M., Alfaro L., Zhang J., Chotiko A., Delgado J.L.B., Chouljenko A., Bankston D., Bechtel P.J., Sathivel S. (2015). Effect of blueberry extract from blueberry pomace on the microencapsulated fish oil. J. Food Process. Preserv..

[21] Böttcher S., Steinhauser U., Drusch S. (2015). Off-flavour masking of secondary lipid oxidation products by pea dextrin. Food Chem..

[22] Galanakis C.M., Tsatalas P., Charalambous Z., Galanakis I.M. (2018). Control of microbial growth in bakery products fortified with polyphenols recovered from olive mill wastewater. Environ. Technol. Innov..

[23] Galanakis C.M., Tsatalas P., Charalambous Z., Galanakis I.M., Charalambous Z. (2018). Polyphenols recovered from olive mill wastewater as natural preservatives in extra virgin olive oils and refined olive kernel oils. Environ. Technol. Innov..

[24] López-Serrano M., Ros Barceló A. (2002). Comparative study of the products of the peroxidase-catalyzed and the polyphenoloxidase-catalyzed (+)-catechin oxidation. Their possible implications in strawberry (Fragaria× ananassa) browning reactions. J. Agric. Food Chem..

[25] Tripodo G., Ibáñez E., Cifuentes A., Gilbert-Lopez B., Fanali C. (2018). Optimization of pressurized liquid extraction by response surface methodology of Goji berry (Lycium barbarum L.) phenolic bioactive compounds. Electrophoresis.

[26] Jiang Y., Duan X., Joyce D., Zhang Z., Li J. (2004). Advances in understanding of enzymatic browning in harvested litchi fruit. Food Chem..

[27] Soto-Vaca A., Gutierrez A., Losso J.N., Xu Z., Finley J.W. (2012). Evolution of Phenolic Compounds from Color and Flavor Problems to Health Benefits. J. Agric. Food Chem..

[28] Caleja C., Barros L., Antonio A.L., Ćirić A., Sokovic M., Oliveira M.B.P., Santos-Buelga C., Ferreira I.C. (2015). Foeniculum vulgare Mill. as natural conservation enhancer and health promoter by incorporation in cottage cheese. J. Funct. Foods.

[29] Peschel W., Sánchez-Rabaneda F., Diekmann W., Plescher A., Gartzía I., Jimenez D., Lamuela-Raventos R.M., Buxaderas S., Codina C. (2006). An industrial approach in the search of natural antioxidants from vegetable and fruit wastes. Food Chem..

[30] Xiu-Qin L., Chao J., Yan-Yan S., Min-Li Y., Xiao-Gang C. (2009). Analysis of synthetic antioxidants and preservatives in edible vegetable oil by HPLC/TOF-MS. Food Chem..

[31] Jeong S.-H., Kim B.-Y., Kang H.-G., Ku H.-O., Cho J.-H. (2005). Effects of butylated hydroxyanisole on the development and functions of reproductive system in rats. Toxicology.

[32] Engin A.B., Bukan N., Kurukahvecioglu O., Memis L., Engin A. (2011). Effect of butylated hydroxytoluene (E321) pretreatment versus l-arginine on liver injury after sub-lethal dose of endotoxin administration. Environ. Toxicol. Pharmacol..

[33] Botterweck A., Verhagen H., Goldbohm R., Kleinjans J., Brandt P.V.D., Brandt P.V.D. (2000). Intake of butylated hydroxyanisole and butylated hydroxytoluene and stomach cancer risk: results from analyses in the Netherlands Cohort Study. Food Chem. Toxicol..

[34] Randhawa S., Bahna S.L. (2009). Hypersensitivity reactions to food additives. Curr. Opin. Allergy Clin. Immunol..

[35] Bleve M., Ciurlia L., Erroi E., Lionetto G., Longo L., Rescio L., Schettino T., Vasapollo G., Lionetto M.G. (2008). An innovative method for the purification of anthocyanins from grape skin extracts by using liquid and sub-critical carbon dioxide. Sep. Purif. Technol..

[36] Kornienko J.S., Smirnova I.S., Pugovkina N.A., Ivanova J.S., Shilina M.A., Grinchuk T.M., Shatrova A.N., Aksenov N.D., Zenin V.V., Nikolsky N.N. (2019). High doses of synthetic antioxidants induce premature senescence in cultivated mesenchymal stem cells. Sci. Rep..

[37] Wang W., Asimakopoulos A.G., Abualnaja K.O., Covaci A., Gevao B., Johnson-Restrepo B., Kumosani T.A., Malarvannan G., Minh T.B., Moon H.-B. (2015). Synthetic Phenolic Antioxidants and Their Metabolites in Indoor Dust from Homes and Microenvironments. Environ. Sci. Technol..

[38] Wang W., Kannan K. (2018). Inventory, loading and discharge of synthetic phenolic antioxidants and their metabolites in wastewater treatment plants. Water Res..

[39] Tajkarimi M., Ibrahim S., Cliver D. (2010). Antimicrobial herb and spice compounds in food. Food Control..

[40] Dickson-Spillmann M., Siegrist M., Keller C. (2011). Attitudes toward chemicals are associated with preference for natural food. Food Qual. Prefer..

[41] Sebranek J., Sewalt V., Robbins K., Houser T. (2005). Comparison of a natural rosemary extract and BHA/BHT for relative antioxidant effectiveness in pork sausage. Meat Sci..

[42] Kobus-Cisowska J., Flaczyk E., Rudzińska M., Kmiecik D. (2014). Antioxidant properties of extracts from Ginkgo biloba leaves in meatballs. Meat Sci..

[43] Abbas M., Saeed F., Anjum F.M., Afzaal M., Tufail T., Bashir M.S., Ishtiaq A., Hussain S., Suleria H.A.R. (2017). Natural polyphenols: An overview. Int. J. Food Prop..

[44] Boschin G., Arnoldi A. (2011). Legumes are valuable sources of tocopherols. Food Chem..

[45] Hussain A., Larsson H., Olsson M.E., Kuktaite R., Grausgruber H., Johansson E. (2012). Is organically produced wheat a source of tocopherols and tocotrienols for health food?. Food Chem..

[46] Omoni A.O., Aluko R.E. (2005). The anti-carcinogenic and anti-atherogenic effects of lycopene: A review. Trends Food Sci. Technol..

[47] Taghvaei M., Jafari S.M. (2015). Application and stability of natural antioxidants in edible oils in order to substitute synthetic additives. J. Food Sci. Technol..

[48] Lorenzo J.M., Pateiro M., Domínguez R., Barba F.J., Putnik P., Kovačević D.B., Shpigelman A., Granato D., Franco D. (2018). Berries extracts as natural antioxidants in meat products: A review. Food Res. Int..

[49] Bártíková H., Skálová L., Valentova K., Matoušková P., Szotáková B., Martin J., Kvita V., Boušová I. (2015). Effect of oral administration of green tea extract in various dosage schemes on oxidative stress status of mice in vivo. Acta Pharm..

[50] Mansour E.H., Khalil A.H. (2000). Evaluation of antioxidant activity of some plant extracts and their application to ground beef patties. Food Chem..

[51] Caleja C., Barros L., Antonio A.L., Carocho M., Oliveira M.B.P., Ferreira I.C. (2016). Fortification of yogurts with different antioxidant preservatives: A comparative study between natural and synthetic additives. Food Chem..

[52] Caleja C., Barros L., Antonio A.L., Oliveira M.B.P., Ferreira I.C. (2017). A comparative study between natural and synthetic antioxidants: Evaluation of their performance after incorporation into biscuits. Food Chem..

[53] Shah M.A., Bosco S.J.D., Mir S.A. (2014). Plant extracts as natural antioxidants in meat and meat products. Meat Sci..

[54] Estévez M., Ventanas S., Cava R. (2006). Effect of natural and synthetic antioxidants on protein oxidation and colour and texture changes in refrigerated stored porcine liver pâté. Meat Sci..

[55] Food and Drug Administration (2000). Guidance for industry and other stakeholders: Toxicological principles for the safety assessment of food ingredients. Redbook.

[56] Lu B., Wu X., Tie X., Zhang Y., Zhang Y. (2005). Toxicology and safety of anti-oxidant of bamboo leaves. Part 1: Acute and subchronic toxicity studies on anti-oxidant of bamboo leaves. Food Chem. Toxicol..

[57] Brondani J.C., Reginato F.Z., Brum E.D.S., Vencato M.D.S., Lhamas C.L., Viana C., Da Rocha M.I.U.M., Bauermann L.D.F., Manfron M.P., Silva C.V. (2017). Evaluation of acute and subacute toxicity of hydroethanolic extract of Dolichandra unguis-cati L. leaves in rats. J. Ethnopharmacol..

[58] Peng K.-Z., Zhang S.-Y., Zhou H.-L. (2016). Toxicological evaluation of the flavonoid-rich extract from Maydis stigma: Subchronic toxicity and genotoxicity studies in mice. J. Ethnopharmacol..

[59] Arantes S.M., Piçarra A., Guerreiro M., Salvador C., Candeias F., Caldeira A.T., Martins M.R. (2019). Toxicological and pharmacological properties of essential oils of Calamintha nepeta, Origanum virens and Thymus mastichina of Alentejo (Portugal). Food Chem. Toxicol..

[60] ANS (2012). Guidance for submission for food additive evaluations. EFSA J..

[61] EFSA (2008). Use of rosemary extracts as a food additive-Scientific Opinion of the Panel on Food Additives, Flavourings, Processing Aids and Materials in Contact with Food. EFSA J..

[62] Bansal S., Choudhary S., Sharma M., Kumar S.S., Lohan S., Bhardwaj V., Syan N., Jyoti S. (2013). Tea: A native source of antimicrobial agents. Food Res. Int..

[63] Dimitrios B. (2006). Sources of natural phenolic antioxidants. Trends Food Sci. Technol..

[64] Jiang J., Xiong Y.L. (2016). Natural antioxidants as food and feed additives to promote health benefits and quality of meat products: A review. Meat Sci..

[65] Freitas A., Moldão-Martins M., Costa H.S., Albuquerque T.G., Valente A., Sanches-Silva A. (2015). Effect of UV-C radiation on bioactive compounds of pineapple (Ananas comosus L. Merr.) by-products. J. Sci. Food Agric..

[66] Fierascu R.C., Ortan A., Fierascu I.C., Fierascu I. (2018). In vitro and in vivo evaluation of antioxidant properties of wild-growing plants. A short review. Curr. Opin. Food Sci..

[67] Halvorsen B.L., Holte K., Myhrstad M.C.W., Barikmo I., Hvattum E., Remberg S.F., Wold A.-B., Haffner K., Baugerød H., Andersen L.F. (2002). A Systematic Screening of Total Antioxidants in Dietary Plants. J. Nutr..

[68] Embuscado M.E. (2015). Spices and herbs: Natural sources of antioxidants—A mini review. J. Funct. Foods.

[69] Yin J., Becker E.M., Andersen M.L., Skibsted L.H. (2012). Green tea extract as food antioxidant. Synergism and antagonism with α-tocopherol in vegetable oils and their colloidal systems. Food Chem..

[70] Fernandes P.A., Le Bourvellec C., Renard C.M., Nunes F.M., Bastos R., Coelho E., Wessel D.F., Coimbra M.A., Cardoso S.M. (2019). Revisiting the chemistry of apple pomace polyphenols. Food Chem..

[71] Brezoiu A.-M., Matei C., Deaconu M., Stanciuc A.-M., Trifan A., Gaspar-Pintiliescu A., Berger D. (2019). Polyphenols extract from grape pomace. Characterization and valorisation through encapsulation into mesoporous silica-type matrices. Food Chem. Toxicol..

[72] Corrêa-Filho L.C., Lourenço S.C., Duarte D.F., Moldão-Martins M., Alves V.D. (2019). Microencapsulation of Tomato (Solanum lycopersicum L.) Pomace Ethanolic Extract by Spray Drying: Optimization of Process Conditions. Appl. Sci..

[73] Panić M., Stojković M.R., Kraljić K., Škevin D., Redovniković I.R., Srček V.G., Radošević K. (2019). Ready-to-use green polyphenolic extracts from food by-products. Food Chem..

[74] Diamanti A.C., Igoumenidis P.E., Mourtzinos I., Yannakopoulou K., Karathanos V.T. (2017). Green extraction of polyphenols from whole pomegranate fruit using cyclodextrins. Food Chem..

[75] Gorinstein S., Martín-Belloso O., Park Y.-S., Haruenkit R., Lojek A., Ĉíž M., Caspi A., Libman I., Trakhtenberg S. (2001). Comparison of some biochemical characteristics of different citrus fruits. Food Chem..

[76] da Silva D.I., Nogueira G.D., Duzzioni A.G., Barrozo M.A. (2013). Changes of antioxidant constituents in pineapple (Ananas comosus) residue during drying process. Ind. Crop. Prod..

[77] George B., Kaur C., Khurdiya D., Kapoor H. (2004). Antioxidants in tomato (Lycopersium esculentum) as a function of genotype. Food Chem..

[78] Perino S., Pierson J.T., Ruiz K., Cravotto G., Chemat F. (2016). Laboratory to pilot scale: Microwave extraction for polyphenols lettuce. Food Chem..

[79] Saffarzadeh-Matin S., Khosrowshahi F.M. (2017). Phenolic compounds extraction from Iranian pomegranate (Punica granatum) industrial waste applicable to pilot plant scale. Ind. Crop. Prod..

[80] Solana M., Mirofci S., Bertucco A. (2016). Production of phenolic and glucosinolate extracts from rocket salad by supercritical fluid extraction: Process design and cost benefits analysis. J. Food Eng..

[81] Naczk M., Shahidi F. (2004). Extraction and analysis of phenolics in food. J. Chromatogr. A.

[82] Azmir J., Zaidul I., Rahman M.M., Sharif K., Mohamed A., Sahena F., Jahurul M., Ghafoor K., Norulaini N., Omar A. (2013). Techniques for extraction of bioactive compounds from plant materials: A review. J. Food Eng..

[83] Marcheafave G.G., Tormena C.D., Pauli E.D., Rakocevic M., Bruns R.E., Scarminio I.S. (2019). Experimental mixture design solvent effects on pigment extraction and antioxidant activity from Coffea arabica L. leaves. Microchem. J..

[84] Casagrande M., Zanela J., Wagner A., Busso C., Wouk J., Iurckevicz G., Montanher P.F., Yamashita F., Malfatti C.R.M. (2018). Influence of time, temperature and solvent on the extraction of bioactive compounds of Baccharis dracunculifolia: In vitro antioxidant activity, antimicrobial potential, and phenolic compound quantification. Ind. Crop. Prod..

[85] Fu Z.-F., Tu Z.-C., Zhang L., Wang H., Wen Q.-H., Huang T. (2016). Antioxidant activities and polyphenols of sweet potato (Ipomoea batatas L.) leaves extracted with solvents of various polarities. Food Biosci..

[86] Socaci S.A., Fărcaș A.C., Diaconeasa Z.M., Vodnar D.C., Rusu B., Tofană M. (2018). Influence of the extraction solvent on phenolic content, antioxidant, antimicrobial and antimutagenic activities of brewers’ spent grain. J. Cereal. Sci..

[87] Do Q.D., Angkawijaya A.E., Tran-Nguyen P.L., Huynh L.H., Soetaredjo F.E., Ismadji S., Ju Y.-H. (2014). Effect of extraction solvent on total phenol content, total flavonoid content, and antioxidant activity of Limnophila aromatica. J. Food Drug Anal..

[88] Boulekbache-Makhlouf L., Medouni L., Medouni-Adrar S., Arkoub L., Madani K. (2013). Effect of solvents extraction on phenolic content and antioxidant activity of the byproduct of eggplant. Ind. Crop. Prod..

[89] Mokrani A., Madani K. (2016). Effect of solvent, time and temperature on the extraction of phenolic compounds and antioxidant capacity of peach (Prunus persica L.) fruit. Sep. Purif. Technol..

[90] Silva L.de., Castelo-Branco V.N., de Carvalho A.G.A., Monteiro M.C., Perrone D., Torres A.G. (2017). Ethanol extraction renders a phenolic compounds-enriched and highly stable jussara fruit (Euterpe edulis M.) oil. Eur. J. lipid Sci. Technol..

[91] Złotek U., Mikulska S., Nagajek M., Świeca M. (2016). The effect of different solvents and number of extraction steps on the polyphenol content and antioxidant capacity of basil leaves (Ocimum basilicum L.) extracts. Saudi J. Biol. Sci..

[92] Li T., Shen P., Liu W., Liu C., Liang R., Yan N., Chen J. (2014). Major Polyphenolics in Pineapple Peels and their Antioxidant Interactions. Int. J. Food Prop..

[93] Al Amri F.S., Hossain M.A. (2018). Comparison of total phenols, flavonoids and antioxidant potential of local and imported ripe bananas. J. Basic Appl. Sci..

[94] Vella F.M., Laratta B., La Cara F., Morana A. (2018). Recovery of bioactive molecules from chestnut (Castanea sativa Mill.) by-products through extraction by different solvents. Nat. Prod. Res..

[95] Lou S.-N., Lai Y.-C., Hsu Y.-S., Ho C.-T. (2016). Phenolic content, antioxidant activity and effective compounds of kumquat extracted by different solvents. Food Chem..

[96] Dhanani T., Shah S., Gajbhiye N., Kumar S. (2017). Effect of extraction methods on yield, phytochemical constituents and antioxidant activity of Withania somnifera. Arab. J. Chem..

[97] Sun C., Wu Z., Wang Z., Zhang H. (2015). Effect of Ethanol/Water Solvents on Phenolic Profiles and Antioxidant Properties of Beijing Propolis Extracts. Evid. Based Complement. Altern. Med..

[98] del Pilar Sánchez-Camargo A., Gutiérrez L.F., Vargas S.M., Martinez-Correa H.A., Parada-Alfonso F., Narváez-Cuenca C.E. (2019). Valorisation of mango peel: Proximate composition, supercritical fluid extraction of carotenoids, and application as an antioxidant additive for an edible oil. J. Supercrit. Fluids.

[99] Ferrentino G., Morozova K., Mosibo O.K., Ramezani M., Scampicchio M. (2018). Biorecovery of antioxidants from apple pomace by supercritical fluid extraction. J. Clean. Prod..

[100] Pereira P., Cebola M.-J., Oliveira M.C., Bernardo-Gil M.G. (2016). Supercritical fluid extraction vs conventional extraction of myrtle leaves and berries: Comparison of antioxidant activity and identification of bioactive compounds. J. Supercrit. Fluids.

[101] Fabrowska J., Ibañez E., Łęska B., Herrero M. (2016). Supercritical fluid extraction as a tool to valorize underexploited freshwater green algae. Algal Res..

[102] Torres-Ossandón M.J., Vega-Gálvez A., López J., Stucken K., Romero J., Di Scala K. (2018). Effects of high hydrostatic pressure processing and supercritical fluid extraction on bioactive compounds and antioxidant capacity of Cape gooseberry pulp (Physalis peruviana L.). J. Supercrit. Fluids.

[103] Suwal S., Perreault V., Marciniak A., Tamigneaux É., Deslandes É., Bazinet L., Jacques H., Beaulieu L., Doyen A. (2019). Effects of high hydrostatic pressure and polysaccharidases on the extraction of antioxidant compounds from red macroalgae, Palmaria palmata and Solieria chordalis. J. Food Eng..

[104] Briones-Labarca V., Giovagnoli-Vicuña C., Cañas-Sarazúa R. (2019). Optimization of extraction yield, flavonoids and lycopene from tomato pulp by high hydrostatic pressure-assisted extraction. Food Chem..

[105] Pinela J., Prieto M., Barros L., Carvalho A.M., Oliveira M.B.P., Saraiva J.A., Ferreira I.C. (2018). Cold extraction of phenolic compounds from watercress by high hydrostatic pressure: Process modelling and optimization. Sep. Purif. Technol..

[106] Briones-Labarca V., Plaza-Morales M., Giovagnoli-Vicuña C., Jamett F. (2015). High hydrostatic pressure and ultrasound extractions of antioxidant compounds, sulforaphane and fatty acids from Chilean papaya (Vasconcellea pubescens) seeds: Effects of extraction conditions and methods. LWT.

[107] Çam M., Yüksel E., Alaşalvar H., Başyiğit B., Şen H., Yılmaztekin M., Ahhmed A., Sağdıç O. (2019). Simultaneous extraction of phenolics and essential oil from peppermint by pressurized hot water extraction. J. Food Sci. Technol..

[108] Mustafa A., Trevino L.M., Turner C. (2012). Pressurized Hot Ethanol Extraction of Carotenoids from Carrot By-Products. Molecules.

[109] Corazza G.O., Bilibio D., Zanella O., Nunes A.L., Bender J.P., Carniel N., Dos Santos P.P., Priamo W.L. (2018). Pressurized liquid extraction of polyphenols from Goldenberry: Influence on antioxidant activity and chemical composition. Food Bioprod. Process..

[110] Abrahão F.R., Rocha L.C.R., Santos T.A., Carmo E.L.D., Pereira L.A.S., Borges S.V., Pereira R.G.F.A., Botrel D.A. (2019). Microencapsulation of bioactive compounds from espresso spent coffee by spray drying. LWT.

[111] Pereira D.T.V., Tarone A.G., Cazarin C.B.B., Barbero G.F., Martínez J. (2019). Pressurized liquid extraction of bioactive compounds from grape marc. J. Food Eng..

[112] Mariotti-Celis M., Martínez-Cifuentes M., Huamán-Castilla N., Vargas-González M., Pedreschi F., Pérez-Correa J. (2018). The Antioxidant and Safety Properties of Spent Coffee Ground Extracts Impacted by the Combined Hot Pressurized Liquid Extraction–Resin Purification Process. Molecules.

[113] Cea Pavez I., Lozano-Sánchez J., Borrás-Linares I., Nuñez H., Robert P., Segura-Carretero A. (2019). Obtaining an Extract Rich in Phenolic Compounds from Olive Pomace by Pressurized Liquid Extraction. Molecules.

[114] Cavalaro R.I., Da Cruz R.G., Dupont S., Bell J.M.L.N.D.M., Vieira T.M.F.D.S. (2019). In vitro and in vivo antioxidant properties of bioactive compounds from green propolis obtained by ultrasound-assisted extraction. Food Chem. X.

[115] López C.J., Caleja C., Prieto M., Barreiro M.F., Barros L., Ferreira I.C. (2018). Optimization and comparison of heat and ultrasound assisted extraction techniques to obtain anthocyanin compounds from Arbutus unedo L. Fruits. Food Chem..

[116] Goula A.M., Ververi M., Adamopoulou A., Kaderides K. (2017). Green ultrasound-assisted extraction of carotenoids from pomegranate wastes using vegetable oils. Ultrason. Sonochemistry.

[117] Bamba B.S.B., Shi J., Tranchant C.C., Xue S.J., Forney C.F., Lim L.-T. (2018). Influence of Extraction Conditions on Ultrasound-Assisted Recovery of Bioactive Phenolics from Blueberry Pomace and Their Antioxidant Activity. Molecules.

[118] Guandalini B.B.V., Rodrigues N.P., Marczak L.D.F. (2019). Sequential extraction of phenolics and pectin from mango peel assisted by ultrasound. Food Res. Int..

[119] Kaderides K., Papaoikonomou L., Serafim M., Goula A.M. (2019). Microwave-assisted extraction of phenolics from pomegranate peels: Optimization, kinetics, and comparison with ultrasounds extraction. Chem. Eng. Process. Process. Intensif..

[120] Alara O.R., Mudalip S.K.A., Abdurahman N.H., Mahmoud M.S., Obanijesu E.O.O. (2019). Data on parametric influence of microwave-assisted extraction on the recovery yield, total phenolic content and antioxidant activity of Phaleria macrocarpa fruit peel extract. Chem. Data Collect..

[121] Chuyen H.V., Nguyen M.H., Roach P.D., Golding J.B., Parks S.E. (2018). Microwave-assisted extraction and ultrasound-assisted extraction for recovering carotenoids from Gac peel and their effects on antioxidant capacity of the extracts. Food Sci. Nutr..

[122] Şahin S., Samli R., Tan A.S.B., Barba F.J., Chemat F., Cravotto G., Lorenzo J.M. (2017). Solvent-Free Microwave-Assisted Extraction of Polyphenols from Olive Tree Leaves: Antioxidant and Antimicrobial Properties. Molecules.

[123] Naczk M., Shahidi F. (2006). Phenolics in cereals, fruits and vegetables: Occurrence, extraction and analysis. J. Pharm. Biomed. Anal..

[124] Chandrasekhar J., Madhusudhan M., Raghavarao K. (2012). Extraction of anthocyanins from red cabbage and purification using adsorption. Food Bioprod. Process..

[125] Płotka-Wasylka J., Rutkowska M., Owczarek K., Tobiszewski M., Namieśnik J. (2017). Extraction with environmentally friendly solvents. TrAC Trends Anal. Chem..

[126] Wang L., Weller C.L. (2006). Recent advances in extraction of nutraceuticals from plants. Trends Food Sci. Technol..

[127] Lapornik B., Prošek M., Wondra A.G. (2005). Comparison of extracts prepared from plant by-products using different solvents and extraction time. J. Food Eng..

[128] Bimakr M., Rahman R.A., Taip F.S., Ganjloo A., Salleh L.M., Selamat J., Hamid A., Zaidul I. (2011). Comparison of different extraction methods for the extraction of major bioactive flavonoid compounds from spearmint (Mentha spicata L.) leaves. Food Bioprod. Process..

[129] Ignat I., Volf I., Popa V.I. (2011). A critical review of methods for characterisation of polyphenolic compounds in fruits and vegetables. Food Chem..

[130] Ju Z.Y., Howard L.R. (2003). Effects of Solvent and Temperature on Pressurized Liquid Extraction of Anthocyanins and Total Phenolics from Dried Red Grape Skin. J. Agric. Food Chem..

[131] Chemat F., Rombaut N., Sicaire A.-G., Meullemiestre A., Fabiano-Tixier A.-S., Abert-Vian M. (2017). Ultrasound assisted extraction of food and natural products. Mechanisms, techniques, combinations, protocols and applications. A review. Ultrason. Sonochemistry.

[132] Li T., Qu X.-Y., Zhang Q.-A., Wang Z.-Z. (2012). Ultrasound-assisted extraction and profile characteristics of seed oil from Isatis indigotica Fort. Ind. Crop. Prod..

[133] Ballard T.S., Mallikarjunan P., Zhou K., O’Keefe S. (2010). Microwave-assisted extraction of phenolic antioxidant compounds from peanut skins. Food Chem..

[134] de Vos P., Faas M.M., Spasojevic M., Sikkema J. (2010). Encapsulation for preservation of functionality and targeted delivery of bioactive food components. Int. Dairy J..

[135] Nedovic V., Kalusevic A., Manojlovic V., Levic S., Bugarski B. (2011). An overview of encapsulation technologies for food applications. Procedia Food Sci..

[136] Gharsallaoui A., Roudaut G., Chambin O., Voilley A., Saurel R. (2007). Applications of spray-drying in microencapsulation of food ingredients: An overview. Food Res. Int..

[137] Estevinho B.N., Rocha F., Santos L., Alves A. (2013). Microencapsulation with chitosan by spray drying for industry applications – A review. Trends Food Sci. Technol..

[138] Kiritsakis K., Goula A.M., Adamopoulos K.G., Gerasopoulos D. (2018). Valorization of Olive Leaves: Spray Drying of Olive Leaf Extract. Waste Biomass Valorization.

[139] Papoutsis K., Golding J.B., Vuong Q., Pristijono P., Stathopoulos C.E., Scarlett C.J., Bowyer M. (2018). Encapsulation of Citrus By-Product Extracts by Spray-Drying and Freeze-Drying Using Combinations of Maltodextrin with Soybean Protein and ι-Carrageenan. Foods.

[140] Fan P., Huber D.J., Su Z., Hu M., Gao Z., Li M., Shi X., Zhang Z. (2018). Effect of postharvest spray of apple polyphenols on the quality of fresh-cut red pitaya fruit during shelf life. Food Chem..

[141] de Souza V.B., Thomazini M., de Carvalho Balieiro J.C., Fávaro-Trindade C.S. (2015). Effect of spray drying on the physicochemical properties and color stability of the powdered pigment obtained from vinification byproducts of the Bordo grape (Vitis labrusca). Food Bioprod. Process..

[142] Beirão-Da-Costa S., Duarte C., Bourbon A.I., Pinheiro A.C., Januário M.I.N., Vicente A.A., Da Costa M.L.B., Delgadillo I. (2013). Inulin potential for encapsulation and controlled delivery of Oregano essential oil. Food Hydrocoll..

[143] Baranauskaite J., Kopustinskiene D.M., Bernatoniene J. (2019). Impact of Gelatin Supplemented with Gum Arabic, Tween 20, and β-Cyclodextrin on the Microencapsulation of Turkish Oregano Extract. Molecules.

[144] Lourenço S.C., Torres C.A., Nunes D., Duarte P., Freitas F., Reis M.A., Fortunato E., Moldão-Martins M., Da Costa L.B., Alves V.D. (2017). Using a bacterial fucose-rich polysaccharide as encapsulation material of bioactive compounds. Int. J. Boil. Macromol..

[145] Burhan A.M., Abdel-Hamid S.M., Soliman M.E., Sammour O.A. (2019). Optimization of the Microencapsulation of Lavender Oil by Spray Drying. J. Microencapsul..

[146] Jiang M., Hong Y., Gu Z., Cheng L., Li Z., Li C. (2019). Preparation of a starch-based carrier for oral delivery of Vitamin E to the small intestine. Food Hydrocoll..

[147] Caleja C., Barros L., Antonio A.L., Ćirić A., Barreira J.C., Sokovic M., Oliveira M.B.P., Santos-Buelga C., Ferreira I.C. (2015). Development of a functional dairy food: Exploring bioactive and preservation effects of chamomile (Matricaria recutita L.). J. Funct. Foods.

[148] Wang S.Y., Gao H. (2013). Effect of chitosan-based edible coating on antioxidants, antioxidant enzyme system, and postharvest fruit quality of strawberries (Fragaria x aranassa Duch.). LWT.

[149] De Dicastillo C.L., Rodríguez F., Guarda A., Galotto M.J. (2016). Antioxidant films based on cross-linked methyl cellulose and native Chilean berry for food packaging applications. Carbohydr. Polym..

[150] Moudache M., Colon M., Nerín C., Zaidi F. (2016). Phenolic content and antioxidant activity of olive by-products and antioxidant film containing olive leaf extract. Food Chem..

[151] Das A.K., Rajkumar V., Nanda P.K., Chauhan P., Pradhan S.R., Biswas S. (2016). Antioxidant Efficacy of Litchi (Litchi chinensis Sonn.) Pericarp Extract in Sheep Meat Nuggets. Antioxidants.

[152] Jayathilakan K., Sharma G., Radhakrishna K., Bawa A. (2007). Antioxidant potential of synthetic and natural antioxidants and its effect on warmed-over-flavour in different species of meat. Food Chem..

[153] Andrés A.I., Petron M.J., Delgado-Adamez J., Lopez M., Timon M. (2017). Effect of tomato pomace extracts on the shelf-life of modified atmosphere-packaged lamb meat. J. Food Process Preserv..

[154] I Rey A., Hopia A., Kivikari R., Kähkönen M. (2005). Use of natural food/plant extracts: cloudberry (Rubus Chamaemorus), beetroot (Beta Vulgaris “Vulgaris”) or willow herb (Epilobium angustifolium) to reduce lipid oxidation of cooked pork patties. LWT.

[155] Alvarez D., Xiong Y., Castillo M., Payne F., Garrido M. (2012). Textural and viscoelastic properties of pork frankfurters containing canola–olive oils, rice bran, and walnut. Meat Sci..

[156] Bodoira R.M., Penci M.C., Ribotta P.D., Martinez M.L. (2017). Chia (Salvia hispanica L.) oil stability: Study of the effect of natural antioxidants. LWT.

[157] Karre L., Lopez K., Getty K.J. (2013). Natural antioxidants in meat and poultry products. Meat Sci..

[158] Massini L., Rico D., Martín-Diana A.B., Barry-Ryan C. (2016). Quality Markers of Functional Tomato Juice with Added Apple Phenolic Antioxidants. Beverages.

[159] Kanatt S.R., Chander R., Sharma A. (2008). Chitosan and mint mixture: A new preservative for meat and meat products. Food Chem..

[160] Yu L., Haley S., Perret J., Harris M. (2002). Antioxidant properties of hard winter wheat extracts. Food Chem..

[161] Fernandez-Lopez J., Sevilla L., Sayas-Barberá E., Navarro C., Marin F., Pérez-Alvarez J. (2003). Evaluation of the Antioxidant Potential of Hyssop (Hyssopus officinalis L.) and Rosemary (Rosmarinus officinalis L.) Extracts in Cooked Pork Meat. J. Food Sci..

[162] Cetin-Karaca H., Newman M.C. (2015). Antimicrobial efficacy of plant phenolic compounds against Salmonella and Escherichia Coli. Food Biosci..

[163] Oswell N.J., Thippareddi H., Pegg R.B. (2018). Practical use of natural antioxidants in meat products in the U.S.: A review. Meat Sci..

[164] Balzan S., Taticchi A., Cardazzo B., Urbani S., Servili M., Di Lecce G., Zabalza I.B., Rodriguez-Estrada M.T., Novelli E., Fasolato L. (2017). Effect of phenols extracted from a by-product of the oil mill on the shelf-life of raw and cooked fresh pork sausages in the absence of chemical additives. LWT.

[165] Özcan M.M., Arslan D. (2011). Antioxidant effect of essential oils of rosemary, clove and cinnamon on hazelnut and poppy oils. Food Chem..

[166] Wibowo S., Vervoort L., Tomic J., Santiago J.S., Lemmens L., Panozzo A., Grauwet T., Hendrickx M., Van Loey A. (2015). Colour and carotenoid changes of pasteurised orange juice during storage. Food Chem..

[167] Caleja C., Ribeiro A., Barros L., Barreira J.C., Antonio A.L., Oliveira M.B.P., Barreiro M.F., Ferreira I.C. (2016). Cottage cheeses functionalized with fennel and chamomile extracts: Comparative performance between free and microencapsulated forms. Food Chem..

[168] Miura Y., Inai M., Honda S., Masuda A., Masuda T. (2014). Reducing Effects of Polyphenols on Metmyoglobin and the in Vitro Regeneration of Bright Meat Color by Polyphenols in the Presence of Cysteine. J. Agric. Food Chem..

[169] Eikelenboom G., Hoving-Bolink A., Kluitman I., Houben J., Klont R. (2000). Effect of dietary vitamin E supplementation on beef colour stability. Meat Sci..

[170] Østerlie M., Lerfall J. (2005). Lycopene from tomato products added minced meat: Effect on storage quality and colour. Food Res. Int..

[171] Ihl M., Silva-Weiss A., Bifani V. (2016). Antioxidant Films and Coatings, in Edible Films and Coatings.

[172] Siripatrawan U., Vitchayakitti W. (2016). Improving functional properties of chitosan films as active food packaging by incorporating with propolis. Food Hydrocoll..

[173] de Moraes Crizel T., de Oliveira Rios A., Alves V.D., Bandarra N., Moldão-Martins M., Flôres S.H. (2018). Active food packaging prepared with chitosan and olive pomace. Food Hydrocoll..

[174] de Moraes Crizel T., de Oliveira Rios A., Alves V.D., Bandarra N., Moldão-Martins M., Flôres S.H. (2018). Biodegradable Films Based on Gelatin and Papaya Peel Microparticles with Antioxidant Properties. Food Bioprocess Technol..

[175] Talón E., Trifkovic K.T., Nedovic V.A., Bugarski B.M., Vargas M., Chiralt A., González-Martínez C. (2017). Antioxidant edible films based on chitosan and starch containing polyphenols from thyme extracts. Carbohydr. Polym..

[176] Adilah Z.M., Jamilah B., Hanani Z.N. (2018). Functional and antioxidant properties of protein-based films incorporated with mango kernel extract for active packaging. Food Hydrocoll..

[177] Nogueira G.F., Fakhouri F.M., de Oliveira R.A. (2019). Effect of incorporation of blackberry particles on the physicochemical properties of edible films of arrowroot starch. Dry. Technol..

[178] Wang L., Guo H., Wang J., Jiang G., Du F., Liu X. (2019). Effects of Herba Lophatheri extract on the physicochemical properties and biological activities of the chitosan film. Int. J. Boil. Macromol..

[179] Rodriguez-Garcia I., Cruz-Valenzuela M.R., Silva-Espinoza B.A., González-Aguilar G.A., Moctezuma E., Gutierrez-Pacheco M.M., Tapia-Rodriguez M.R., Ortega-Ramirez L.A., Ayala-Zavala J.F. (2016). Oregano (Lippia graveolens) essential oil added within pectin edible coatings prevents fungal decay and increases the antioxidant capacity of treated tomatoes. J. Sci. Food Agric..

[180] Noori S., Zeynali F., Almasi H. (2018). Antimicrobial and antioxidant efficiency of nanoemulsion-based edible coating containing ginger (Zingiber officinale) essential oil and its effect on safety and quality attributes of chicken breast fillets. Food Control..

[181] Ventura-Aguilar R., Bautista-Baños S., Flores-García G., Zavaleta-Avejar L. (2018). Impact of chitosan based edible coatings functionalized with natural compounds on Colletotrichum fragariae development and the quality of strawberries. Food Chem..

[182] Choulitoudi E., Ganiari S., Tsironi T., Ntzimani A., Tsimogiannis D., Taoukis P., Oreopoulou V. (2017). Edible coating enriched with rosemary extracts to enhance oxidative and microbial stability of smoked eel fillets. Food Packag. Shelf Life.

[183] Choulitoudi E., Bravou K., Bimpilas A., Tsironi T., Tsimogiannis D., Taoukis P., Oreopoulou V. (2016). Antimicrobial and antioxidant activity of Satureja thymbra in gilthead seabream fillets edible coating. Food Bioprod. Process..

[184] Murmu S.B., Mishra H.N. (2017). Optimization of the arabic gum based edible coating formulations with sodium caseinate and tulsi extract for guava. LWT.

[185] Dong F., Wang X. (2018). Guar gum and ginseng extract coatings maintain the quality of sweet cherry. LWT.

[186] Qin Y.Y., Zhang Z.H., Li L., Yuan M.L., Fan J., Zhao T.R. (2015). Physio-mechanical properties of an active chitosan film incorporated with montmorillonite and natural antioxidants extracted from pomegranate rind. J. Food Sci. Technol..

[187] do Rosário Martins M., Arantes S., Candeias F., Tinoco M.T., Cruz-Morais J. (2014). Antioxidant, antimicrobial and toxicological properties of Schinus molle L. essential oils. J. Ethnopharmacol..

[188] Yuan G., Lv H., Yang B., Chen X., Sun H. (2015). Physical Properties, Antioxidant and Antimicrobial Activity of Chitosan Films Containing Carvacrol and Pomegranate Peel Extract. Molecules.

